# Sulforaphane Alters β-Naphthoflavone-Induced Changes in Activity and Expression of Drug-Metabolizing Enzymes in Rat Hepatocytes

**DOI:** 10.3390/molecules22111983

**Published:** 2017-11-16

**Authors:** Kateřina Lněničková, Andrea Dymáková, Barbora Szotáková, Iva Boušová

**Affiliations:** Department of Biochemical Sciences, Faculty of Pharmacy, Charles University, Heyrovského 1203, 50005 Hradec Králové, Czech Republic; lnenickk@faf.cuni.cz (K.L.); dymakoan@faf.cuni.cz (A.D.); szotakova@faf.cuni.cz (B.S.)

**Keywords:** sulforaphane, β-naphthoflavone, drug-metabolizing enzymes, cytochrome P450, NAD(P)H:quinone oxidoreductase 1, glutathione S-transferase, enzyme activity, gene expression

## Abstract

Sulforaphane (SFN), an isothiocyanate found in cruciferous vegetables, exerts many beneficial effects on human health such as antioxidant, anti-inflammatory, and anticancer effects. The effect of SFN alone on drug-metabolizing enzymes (DMEs) has been investigated in numerous in vitro and in vivo models, but little is known about the effect of SFN in combination with cytochrome P450 (CYP) inducer. The aim of our study was to evaluate the effect of SFN on the activity and gene expression of selected DMEs in primary cultures of rat hepatocytes treated or non-treated with β-naphthoflavone (BNF), the model CYP1A inducer. In our study, SFN alone did not significantly alter the activity and expression of the studied DMEs, except for the glutathione S-transferase (GSTA1) mRNA level, which was significantly enhanced. Co-treatment of hepatocytes with SFN and BNF led to a substantial increase in sulfotransferase, aldoketoreductase 1C, carbonylreductase 1 and NAD(P)H:quinone oxidoreductase 1 activity and a marked decrease in cytochrome P450 (CYP) *Cyp1a1*, *Cyp2b* and *Cyp3a4* expression in comparison to the treatment with BNF alone. Sulforaphane is able to modulate the activity and/or expression of DMEs, thus shifting the balance of carcinogen metabolism toward deactivation, which could represent an important mechanism of its chemopreventive activity.

## 1. Introduction

The consumption of cruciferous vegetables (e.g., broccoli, cabbage, Brussels sprouts, and cauliflower) may reduce the risk of cancer and neurodegenerative disease development, as has been evidenced in a number of recent epidemiological studies [1,2]. These beneficial effects of cruciferous vegetables are ascribed to the sulfur- and nitrogen-containing phytochemical glucosinolates, which are hydrolyzed to the bioactive isothiocyanates (ITCs) by the plant enzyme myrosinase [3].

One of the most studied ITCs is sulforaphane (SFN; 4-methylsulfinylbutyl isothiocyanate), which, in cruciferous vegetables (mainly in broccoli), is present in the form of glucosinolate glucoraphanin. After consumption, the degradation of glucoraphanin starts during chewing, in the presence of the plant enzyme myrosinase, and is mediated by the β-thioglucosidases present in human colon microflora as well [3]. Due to its size and lipophilicity, SFN molecules easily diffuse into enterocytes and its plasmatic concentration reaches a micromolar level [4]. This compound is able to cross the blood–brain barrier and accumulate in cerebral tissues. Upon absorption, SFN is conjugated with glutathione by glutathione S-transferase (GST) and metabolized via the mercapturic acid pathway to be excreted predominantly as *N*-acetylcysteine conjugates [5].

The described mechanisms underlying the cancer chemopreventive effects of ITCs are multiple and include the induction of apoptosis and cell cycle arrest, the inhibition of angiogenesis, the modulation of phase I and phase II drug-metabolizing enzymes (DME), and their anti-inflammatory effects [6,7]. The modulatory effects of SFN and other ITCs on various DMEs have been studied in numerous in vitro and in vivo models, e.g., in human volunteers [8], laboratory animals [9], various cell lines and subcellular fractions [7]. The modulation of DMEs is probably caused by the inhibition of the catalytic activity of various cytochrome P450 (CYP) enzymes, which can activate certain carcinogens, and by the induction of some antioxidant and phase II enzymes’ expression [7,10]. The induction of phase II enzymes by SFN is associated with the modification of the thiol group of Keap1, leading to the disruption of Nrf2-Keap1 interactions and increased nuclear translocation of the transcription factor Nrf2 (nuclear factor E2-related protein). Once free from Keap1, Nrf2 dimerizes with small Maf proteins and binds to antioxidant/electrophile response elements (ARE/EpRE) found in the promoter regions of numerous phase II/antioxidant genes [11,12].

Although the effect of SFN alone on DMEs has been investigated in numerous in vitro and in vivo models, little is known about the effect of SFN in combination with CYP inducer. The aim of present study was to evaluate the effect of SFN on the activity and mRNA expression of selected DMEs in primary rat hepatocytes, which were treated or non-treated with the model CYP1A inducer β-naphthoflavone (BNF). Two different schemes of SFN and BNF administration were applied: (1) co-administration of SFN and BNF and (2) pre-treatment of hepatocytes with BNF before SFN administration. The studied enzymes include four isoforms of cytochromes P450 (CYPs), carbonyl-reducing enzymes (NAD(P)H:quinone oxidoreductase 1, NQO1; carbonylreductase, CBR; aldoketoreductase 1C, AKR1C), and conjugation enzymes (GST; sulfotransferase, SULT; UDP-glucuronosyltransferase, UGT).

## 2. Results

The DME activities and corresponding mRNA expressions were determined in subcellular fractions obtained from rat hepatocytes. Drug-metabolizing enzymes in rat hepatocytes were designed as human orthologues because the substrates specific for individual human enzymes/isoforms were used in enzyme assays. First, SFN cytotoxicity was tested in the primary rat hepatocytes and this compound was non-toxic in the concentration range 1–100 μM (data not shown).

### 2.1. Effects of SFN on Cytochrome P450 Enzymes

Hydroxylation or *O*-deethylation of four substrates designed for orthologous human CYP forms and selectively catalyzed by individual rat CYP isoforms, were assayed in hepatic microsomes of control as well as SFN- and/or BNF-treated groups.

In rat hepatocytes, ethoxyresorufin *O*-deethylase activity (EROD; specific for CYP1A2 and CYP2C6) was detected only in groups containing BNF, in which CYP activity was induced by this compound. Compared to the BNF group, SFN inhibited EROD activity by 42.6% and 39.1% in the BNF + SFN and BNF→SFN group, respectively. Specific activities of methoxyresorufin *O*-deethylase MROD (CYP1A2), benzyloxyresorufin *O*-deethylase BROD (CYP1B1 and CYP1A2) and midazolam 1′-hydroxylation (CYP3A) were not detected.

**Figure 1:**
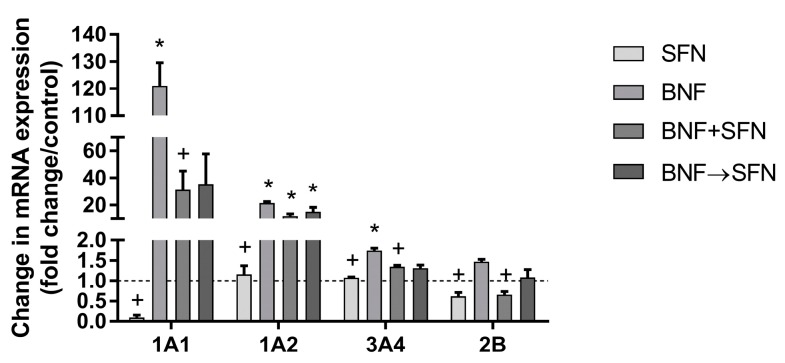
Effect of sulforaphane (10 μM) on the mRNA expression of individual isoforms of cytochrome P450 in rat hepatocytes treated or non-treated with β-naphthoflavone (10 μM). Primary rat hepatocytes were incubated at 37 °C for 24 h. The data are expressed as the mean ± SD (*n* = 3). The mRNA significantly altered (*p* ≤ 0.01) compared to control (*) or β-naphthoflavone (+). BNF β-naphthoflavone; SFN sulforaphane; BNF + SFN co-administration of BNF + SFN; BNF→SFN 6-h BNF pre-treatment followed by SFN addition.

The quantification of individual rat CYP mRNA in hepatocytes showed significant enhancement in *Cyp1a1*, *Cyp1a2* and *Cyp3a4* in the BNF group compared to control. The most pronounced BNF-mediated induction was seen in *Cyp1a1*, where a 120-fold increase in mRNA expression was detected. While SFN alone did not influence the mRNA expression of selected CYPs, this compound significantly decreased the BNF-induced expression of *Cyp2b1* and *Cyp3a4* in the BNF + SFN group (Figure 1).

### 2.2. Effects of SFN on Carbonyl-Reducing Enzymes

The activities and mRNA expression of three types of xenobiotic reductases (AKR1A, CBR1 and NQO1) were determined in cytosolic fractions obtained from rat hepatocytes. The activities toward relatively specific substrates were found and the obtained results were compared among individual groups.

**Figure 2:**
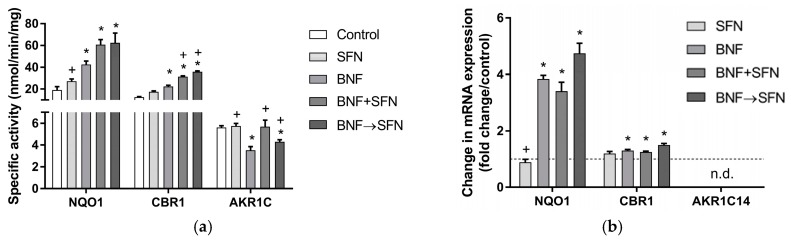
Effect of sulforaphane (10 μM) on the activity (**a**) and mRNA expression (**b**) of carbonyl-reducing enzymes in rat hepatocytes treated or non-treated with β-naphthoflavone (10 μM). Primary rat hepatocytes were incubated at 37 °C for 24 h. All enzyme activities were assessed in the cytosolic fraction. Specific enzyme activities of NADPH-quinone oxidoreductase (NQO1), carbonyl reductase 1 (CBR1) and aldo-keto reductase 1C (AKR1C) were determined as nmol of formed product (NQO1, AKR1C) or consumed cofactor (CBR1) per min per mg of protein. The data are expressed as the mean ± SD (*n* = 3). The mRNA and enzyme activity significantly altered (*p* ≤ 0.01) compared to control (*) or β-naphthoflavone (+). BNF β-naphthoflavone; SFN sulforaphane; BNF + SFN co-administration of BNF + SFN; BNF→SFN 6-h BNF pre-treatment followed by SFN addition; n.d. not determined.

Specific activities of the studied carbonyl-reducing enzymes were unaffected by SFN. Conversely, BNF treatment caused a significant increase in NQO1- and CBR1-specific activities and a marked reduction in the specific activity of AKR1C. In the BNF + SFN and BNF→SFN groups, specific activities of NQO1 and CBR1 were raised even more than in the BFN group, which suggests a probable synergistic effect of SFN and BNF. In the case of AKR1C, the negative effect of BNF on its catalytic activity was reversed by SFN in the BNF + SFN and BNF→SFN groups (Figure 2A).

The expression of *Nqo1* was significantly induced by BNF, while SFN had no effect. In the BNF + SFN and BNF→SFN groups, the effect of SFN on BNF-induced *Nqo1* expression was negligible. The induction of *Cbr1* expression was mild but significant in all treated groups containing BNF (Figure 2B). The expression of *Akr1c14* (also known as *Akr1c9*) was not detected and the observed changes in the AKR1C activities should be therefore ascribed to other AKR isoforms.

### 2.3. Effects of SFN on Conjugation Enzymes

The activity and mRNA expression of the three types of conjugating enzymes were determined in microsomal (UGT) and cytosolic (GST, SULT) fractions obtained from rat hepatocytes. 

**Figure 3:**
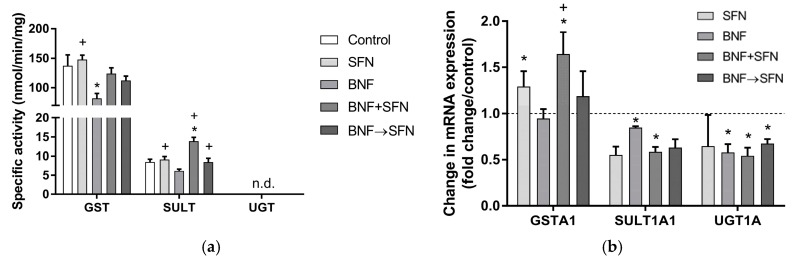
Effect of sulforaphane (10 μM) on the activity (**a**) and mRNA expression (**b**) of conjugation enzymes in rat hepatocytes treated or non-treated with β-naphthoflavone (10 μM). Primary rat hepatocytes were incubated at 37 °C for 24 h. Specific enzyme activities of glutathione S-transferases (GST; cytosol), sulfotransferases (SULT; cytosol) and UDP-glucuronosyl transferases (UGT; microsomes) were determined as nmol of formed product (GST, SULT) or consumed substrate (UGT) per min per mg of protein. The data are expressed as the mean ± SD (*n* = 3). The mRNA or specific activity significantly altered (*p* ≤ 0.01) compared to control (*) or β-naphthoflavone (+). BNF β-naphthoflavone; SFN sulforaphane; BNF + SFN co-administration of BNF + SFN; BNF→SFN 6-h BNF pre-treatment followed by SFN addition; n.d. not determined.

In the rat hepatocytes, the enzymatic activity of UGT was undetectable, while that of GST and SULT was found in these cells. The specific activity of GST and SULT was not influenced by SFN, while BNF inhibited both enzymes, although this inhibition was significant only in the case of GST. On the other hand, SULT-specific activity was markedly increased in the hepatocytes co-treated with BNF and SFN. SFN caused a significant rise in SULT activity in the BNF + SFN and BNF→SFN groups, while the increase in GST activity observed in these groups was insignificant (Figure 3A).

The elevation in *Gsta1* expression was significant in the SFN and BNF + SFN groups, while the effect of BNF alone was negligible. Co-treatment of BNF with SFN caused a marked increase in *Gsta1* transcript levels compared to BNF. The levels of *Sult1a1* and *Ugt1a* transcripts were decreased in all groups compared to the control, although this decline was statistically significant only in the BNF and BNF + SFN groups in the case of *Sult1a1,* and in the BNF, BNF + SFN and BNF→SFN groups in the case of *Ugt1a* (Figure 3B).

## 3. Discussion

In this study, the ability of SFN and BNF to modulate selected phase I and phase II DMEs on the transcriptional and catalytic levels was investigated in primary rat hepatocytes. Hepatocyte cultures are a widely accepted in vitro tool to evaluate mechanisms of drug uptake and metabolism, as well as cytochrome P450 induction potential. Primary hepatocytes possess a complete intracellular machinery and physiological concentrations of cofactors for phase I and phase II reactions, and a complete array of transporters and nuclear receptors, which allow the proper regulation and induction of enzymes and transport proteins [13].

Cytochromes P450, phase I DMEs, have been implicated in the bioactivation of carcinogens. Thus, compounds that could regulate mRNA transcript levels or the activity of CYPs, are thought to be important for the prevention of chemical-induced carcinogenesis [7]. Numerous studies dealt with the modulatory effects of SFN on the activity, protein and gene expression of CYPs in vitro as well as in vivo. In primary rat hepatocytes, SFN significantly inhibited CYP1A1/2, CYP3A and CYP2B activities after 24-h treatment [6,14]. In human hepatocytes, SFN caused a marked decrease in CYP2A6, CYP2C9, CYP2D6, and CYP3A4 activity, but the protein expression of these forms remained unchanged, which indicates that the SFN-caused decrease in CYP activity was not due to the down-regulation of CYP protein [15]. Ten-day oral treatment with SFN (3 mg/kg or 12 mg/kg) caused a significant decrease in rat liver CYP1A2, CYP2B, and CYP3A4 activity, while the protein expression of CYP1A1/2 was markedly elevated and that of CYP2B and CYP3A2 was decreased [16]. Conversely, no change in hepatic CYP1A2 activity was observed in SFN-treated rats [17]. In our experiments, CYP1A2 activity was detectable only upon induction with BNF, where SFN caused a significant decrease in BNF-induced CYP1A2 activity, which is in accordance with previously published results [16]. 

Our results show that SFN does not induce mRNA expression of studied CYPs (Figure 1). Similarly, no changes in the mRNA transcript levels of *Cyp1a1* and *Cyp1a2* were found in SFN-treated rat hepatocytes [6]. In primary human hepatocytes treated with SFN 10 μM, no induction in the mRNA expression of *Cyp1a1*, *Cyp1a2*, *Cyp2b6*, *Cyp2c9* and *Cyp3a4* after 24-h treatment was observed [15], while a great decrease in *Cyp3a4* mRNA after 48-h treatment was reported [18]. In our experiments, the treatment of rat hepatocytes with BNF led to *Cyp1a1*, *Cyp1a2*, and *Cyp3a* mRNA induction, which was diminished by SFN co-administration (Figure 1). The expression of individual CYP families is regulated via various nuclear receptors, including the aryl hydrocarbon receptor (AhR; CYP1A), pregnane X-receptor (PXR; CYP3A and CYP2B) and constitutive androstane receptor (CAR; CYP2B), which can be activated by numerous ligands including BNF, benzo[a]pyrene (both AhR), rifampicin (PXR), and phenobarbital (CAR). SFN acted as an effective in vitro antagonist of rat AhR and human PXR/CAR, with a subsequent decrease in benzo[a]pyrene-induced *Cyp1a1* and rifampicin-induced *Cyp3a4* and *Cyp2b6* expression [19,20,21]. 

Among phase I DMEs, the most studied carbonyl-reducing enzyme in relation to SFN is NAD(P)H:quinone oxidoreductase 1 (NQO1, also known as quinone reductase), which is recognized as an antioxidant, chemoprotective, and cancer-preventive enzyme. The catalytic activity of NQO1 was increased upon SFN treatment in rats [16], neonatal rat cardiomyocytes [22], murine hepatoma Hepa1c1c7, and human prostate cancer LNCaP cells [23]. Our data showed only a mild, insignificant increase in NQO1 activity and no change in *Nqo1* expression in the SFN group compared to the control (Figure 2). In SFN-treated mice, the mRNA and protein expression of NQO1 was raised three times and 1.9 times compared to the control, respectively [24]. The induction of NQO1 at the activity and transcriptional levels was due to the SFN-mediated Nrf2 activation [6,25]. In our study, BNF caused significant induction of NQO1 activity as well as mRNA levels. In accordance with our results, BNF acted as an NQO1 activity inducer in murine hepatoma Hepa1c1c7, human hepatoma HepG2, human breast cancer MCF7, and human prostate cancer LNCaP cells [23]. In our experiments, synergistic effects of SFN and BNF on NQO1 activity and mRNA expression were observed in the BNF + SFN and BNF→SFN groups (Figure 2). This synergistic effect could be explained by direct Nrf2 activation by SFN and its transactivation by a hydroxylated metabolite of BNF. Bifunctional inducers (such as BNF) are AhR ligands and upon induction of CYP1A1/2 and the subsequent oxidation of this xenobiotic by CYP, the hydroxylated metabolite is able to transactivate Nrf2-traget genes in an AhR-independent manner [11].

The influence of SFN on the activity/expression of other phase I carbonyl-reducing enzymes was studied to a much lower extent. In our experiments, no changes in the specific activity of AKR1C were found in hepatocytes treated with SFN. Conversely, nontoxic doses of SFN (50 μM) caused an increase in the catalytic activity, protein and mRNA levels of AKR1C1 in human colorectal adenocarcinoma Caco-2 cells [25]. This discrepancy is probably caused by the five times lower concentration of SFN and the different model used in our experiments. The effect of SFN on AKR1A1 activity differed among various cell lines; it was unchanged in Hepa1c1c7, MCF-7 and HepG2 cells, while activity in LNCaP cells was elevated [23]. BNF-treatment exerted contradictory effects on AKR1A1 in various cell lines; AKR1A1 activity was decreased by 40% in MCF-7 cells, while it was increased in Hepa1c1c7 and HeLa cells [23]. Treatment with BNF achieved a significant induction in AKR1C1 activity in Caco-2 lines [25], while our results showed a marked decline in AKR1C activity caused by BNF, which was reversed by co-treatment with SFN (Figure 2). To our knowledge, this is the first time that the effects of SFN on the activity and expression of CBR1 are reported. In our experiments, the induction of CBR1 activity and mRNA expression upon SFN and BFN treatment were observed. Accordingly, a strong BNF-mediated induction in *Cbr1* expression was reported in HepG2 and MCF-7 cells [26]. As in the case of NQO1, SFN and BNF have synergistic effects on CBR1 activity, which could be explained by the direct Nrf2 activation by SFN and its transactivation by a hydroxylated metabolite of BNF [11]; these were observed in the BNF + SFN and BNF→SFN groups (Figure 2). 

One of the most-studied phase II DMEs regarding SFN is glutathione S-transferase (GST), which represents one of the important cell protective mechanisms against chemical toxicity and oxidative stress [11]. Contradictory results regarding SFN’s effect on GST activity have been found in the literature. The influence of SFN on GST activity differed among various cell lines: a significant rise was reported in Hep1c1c7 and LNCaP cells, while a marked decrease was observed in HepG2 and HT-29 cells and no changes were found in MCF-7 and HeLa cells [24]. In rat hepatocytes, SFN-treatment caused a 1.5-fold increase in GST activity [6], while no change in GST activity was observed in our experiments (Figure 3A). This discrepancy could be explained by the lower SFN concentration used in our work (40 μM vs. 10 μM). In accordance, no changes in GST activity were observed in vivo in rats administered by SFN at the dose 3 mg/kg and 12 mg/kg [16]. Interestingly, a BNF-mediated decrease in GST activity was observed in our experiments, although the mRNA levels of *Gsta1* remained unchanged. Similarly, previously published results on MCF-7 cells showed a decline in GST activity upon incubation with BNF [23]. On the other hand, BNF treatment mediated an increase in GST activity in Hepa1c1c7 and LNCap cells [23] and in porcine hepatic subcellular fractions [27]. The observed decrease was partially reversed by the co-administration of SFN (Figure 3A). Only non-significant changes in *Gsta1* mRNA expression were observed in the BNF and BNF→SFN groups, while SFN alone as well as the co-treatment of SFN with BNF caused a marked increase in *Gsta1* expression (Figure 3). Accordingly, a dose-dependent SFN-mediated *Gsta1/2* induction was described in primary rat hepatocytes [28]. On the other hand, BNF treatment caused a marked inhibition of *Gsta1* and *Gsta2* expression in undifferentiated HepaRG cells [11]. In male 129/sv mice, SFN induced mRNA and protein expression of GSTA4, while those of GSTA1/2, GSTA3 and GSTM1 remained unchanged [24]. In mouse embryonic fibroblasts, SFN treatment caused Nrf2-mediated induction of GSTA4, GSTM1 and GSTP1 protein and gene expressions [11].

Only a little is known about SFN and BNF’s effects on the activity and/or expressions of UDP-glucuronosyltransferase (UGT) and sulfotransferase (SULT), two important DMEs catalyzing conjugation reactions. In our experiments, the inhibition of *Ugt1a1* expression was found in all groups, although the inhibition observed in the SFN group was insignificant (Figure 3B). Accordingly, treatment with SFN had no effect on UGT activity in rats [16] and on *Ugt1a1* expression in differentiated Caco-2 cells [8]. In male mice, BNF treatment caused an induction in hepatic *Ugt1a1*, *Ugt1a5*, *Ugt1a6* and *Ugt1a9* and a significant inhibition in *Ugt2a3*, while the transcriptions of *Ugt1a2*, *Ugt1a7*, *Ugt1a10*, *Ugt2b5*, *Ugt2b34*, *Ugt2b25* and *Ugt2b36* were not altered [29]. To our knowledge, this is the first report on SFN’s effects on hepatic SULT activity and mRNA expression. Interestingly, co-treatment of SFN with BNF caused a substantial increase in SULT activity, while SFN alone and BNF treatment followed by SFN did not change SULT activity and BNF alone caused a negligible inhibition in comparison to the control. Moreover, SFN was able to counteract the negative effect of BNF on SULT activity in both groups containing SFN and BNF (Figure 3A). The gene expression of *Sult1a1* was reduced in all groups compared to the control, but this inhibition was significant only in BNF and BNF + SFN groups (Figure 3B). The discrepancy between the results obtained on SULT activity and mRNA expression could be explained by the fact that measurement of phenol SULT activity might result from the activity of several other SULT1 isoforms (e.g., SULT1A1, SULT1A3, SULT1B1, SULT1C2, and SULT1E1) that also utilize 2-naphthol [30].

The weak correlation among the mRNA expression levels and activities of CBR1, GST and SULT suggests a substantial role for regulatory post-transcriptional, translational and post-translational processes. Most reports on mRNA and protein levels find only a weak correlation between the respective abundances of these two classes of biological molecules. Several biological factors influencing this correlation were identified [31]. Post-transcriptional gene regulation is also mediated by microRNAs, which are able to suppress mRNA translation or accelerate its degradation [32]. On the other hand, good correlation between the induction of the CYP1A2, CYP2B6 and CYP3A4 enzyme activities and mRNA expression in rat and human hepatocytes treated with prototypical inducers was described in [33]. The induction of CYP1A1/2 by BNF caused a gradual increase in mRNA, the corresponding protein levels and enzyme activity in rat hepatocytes during 24-h treatment (all parameters were assayed at the times 2 h, 4 h, 12 h and 24 h), with good correlation of all tested parameters. In the case of CYP1A1/2, any time interval and any parameter seemed to be suitable for an induction study. A different response was obtained for NQO1 and GSTA upon BNF treatment, with low correlation of the studied parameters [34]. From the pharmacological/toxicological point of view, enzyme activity is the most relevant marker, since this ultimately affects the clearance of a metabolized drug. However, when the induction study is performed only at one time-point, the induction effect can be underestimated and some valuable information can be missed. Therefore, the concomitant testing of at least two parameters is better for a more accurate interpretation of the induction study results.

## 4. Materials and Methods

### 4.1. Chemicals

Acenaphthenol, benzyloxyresorufin, bicinchoninic acid (BCA) assay kit, cytochrome c, NADH, NADPH, NADP^+^, 7-ethoxyresorufine, menadione, 7-methoxyresorufine, β-naphthoflavone, *p*-nitrophenyl sulfate, resorufin, *R*-sulforaphane, 3′-phosphoadenosin-5′-phosphate, and UDP-glucuronic acid were purchased from Sigma-Aldrich (Prague, Czech Republic). Midazolam was obtained from Toronto Research Chemicals (North York, ON, Canada). All other chemicals used were of HPLC or analytical grade. For real-time Polymerase Chain Reaction (PCR) analyses, ProtoScript^®^ II Reverse Transcriptase was purchased from NEB (Ipswich, UK), TriReagent from Biotech (Prague, Czech Republic), and the qPCRCore kit for SYBR Green I from Eurogentec (Seraing, Belgium).

### 4.2. Isolation and Culture of Rat Hepatocytes

Male Wistar rats were obtained from Meditox (Konárovice, Czech Republic). They were housed in air-conditioned animal quarters with a 12 h light/dark cycle. Food (a standard rat chow diet) and water were provided ad libitum. The rats were cared for and used in accordance with the Guide for the Care and Use of Laboratory Animals (Protection of Animals from Cruelty Act No. 246/92, Czech Republic). Rats were sacrificed by decapitation and livers were removed immediately. Hepatocytes were isolated by a two-step collagenase method from rat liver [35]. Isolated hepatocytes were mixed with culture medium ISOM (1:1 mixture of Ham F12 and Williams′ E). The viability of hepatocytes was determined using Trypan blue. One million viable cells in 3 mL of culture medium were placed into Petri dishes (6 cm in diameter). Fetal calf serum was added to the culture medium (5%) during the first 4 h to favor the attachment of cells. After that, the medium was changed with medium containing tested compounds. Five sets of samples were prepared: (1) control (0.1% DMSO); (2) SFN 10 μM; (3) BNF 10 μM; (4) co-administration of SFN + BNF (both 10 μM); 5) pre-treatment with BNF 10 μM for 6 h followed by SFN 10 μM for next 18 h (group BNF→SFN). The cultures were maintained at 37 °C in a humid atmosphere of air with 5% CO_2_ for 24 h. 

### 4.3. Preparation of Subcellular Fractions

Microsomal and cytosolic fractions were obtained from control and treated hepatocytes. Cells were homogenized using sonication with Sonopuls (Bandelin, Germany). The subcellular fractions were isolated by differential centrifugation of the cell homogenate (20,000 *g*, 60 min, 4 °C) and the supernatant was further centrifuged (105,000 *g*, 60 min, 4 °C). Supernatant and pellet correspond to cytosol and microsomes, respectively. The microsomes were suspended in 0.1 M sodium phosphate buffer (pH 7.4) containing 20% glycerol (*v*/*v*). Subcellular fractions were stored at −80 °C.

### 4.4. RNA Isolation and Quantitative Real-Time PCR (qPCR)

First, the total RNA was isolated using TriReagent according to the manufacturer’s instructions (Biotech, Prague, Czech Republic). RNA concentration and purity was detected spectrophotometrically at 260 and 280 nm using a NanoDrop ND-1000 UV-Vis Spectrophotometer (Thermo Scientific, Waltham, MA, USA). ProtoScript II reverse transcriptase and oligo(dT)18 were used to perform the first-strand complementary DNA synthesis. qPCR analyses were done in a QuantStudio 6 thermocycler (Applied Biosystems, Thermo Fisher Scientific, Waltham, MA, USA) using a qPCR Core kit for SYBR^TM^ Green I following the manufacturer’s protocol. The used primers were designed manually and are presented in Table 1. Gene expression was determined in three independent samples. The relative mRNA levels were normalized to the reference gene glyceraldehyde 3-phosphate dehydrogenase (GAPDH), which stability upon BNF and SFN treatment was verified. The gene expression calculations were based on the Delta–Delta Ct method [36] and the results were expressed as the fold change of the treated groups relative to the control (DMSO). 

### 4.5. Enzyme Assays

Enzyme activities were assessed in cytosolic and microsomal fractions prepared from control and treated hepatocytes. The enzyme assays (each performed in 4–8 replicates) were repeated three times. The amount of organic solvents in the final reaction mixtures did not exceed 1% (*v*/*v*). Enzyme catalytic activity was normalized to mg of protein in cytosolic or microsomal fractions. Protein concentration in subcellular fractions was determined using the bicinchoninic acid (BCA) assay according to manufacturer’s protocol (Sigma-Aldrich, Prague, Czech Republic).

The enzyme activities of individual CYP forms were measured according to [37] and [38] with slight modification; the activity was measured for 30 min at 37 °C. The following microsomal CYP activities were assayed: ethoxyresorufin-*O*-deethylase (EROD), relatively specific for rat CYP1A2 and CYP2C6 [39]; methoxyresofin-*O*-deethylase (MROD), relatively specific for CYP1A2 [40], and benzoxyresorufin-*O*-deethylase (BROD), specific for CYP2B1 and CYP1A2 [39]. Each reaction mixture contained 0.1 M Tris HCl (pH 7.4), 5 mM MgCl_2_, 2.5 μM substrate (ethoxyresorufin, methoxyresorufin or benzoxyresorufin), 500 μM NADPH and 5 μL of microsomes. The arising fluorescence of resorufin was measured using the microplate reader Tecan Infinite M200 (Tecan Group; Mannedorf, Switzerland). Activity of CYP3A was determined as midazolam 1′-hydroxylation [41] using the HPLC system (Shimadzu, Kyoto, Japan). Briefly, a reaction mixture containing 0.1 M Na-phosphate buffer (pH 7.8), 100 μM midazolam, 1 mM NADPH, 2 mM MgCl_2_ and 40 μL of microsomes was incubated for 30 min at 37 °C. The reaction was stopped by 50 μL of 2.5 M NaOH. After the addition of 1 μg of internal standard (diazepam), samples were extracted to ethyl acetate (2 min), centrifuged (10,000 *g*, 15 min) and evaporated (30 °C). Prior to the HPLC analyses, samples were dissolved in mobile phase consisting of 0.05 M Na_2_HPO_4_ (pH 4.5) adjusted with phosphoric acid and acetonitrile mixture (67:33). Activities were measured using the Prominence HPLC system (Shimadzu, Kyoto, Japan), equipped with a LiChroCART 250-4 LiChrospher 100 RP-18 column (Merck, Darmstadt, Germany) and UV detection. Absorbance was monitored at 227 nm.

All other enzyme assays were based on the spectrophotometric detection of either the product formed or the detection of decreasing substrate/cofactor levels in cytosolic or microsomal fractions using the microplate reader Tecan Infinite M200 (Tecan Group; Mannedorf, Switzerland). All enzyme activities were determined using slight modifications of previously published methods. 

The activity of the carbonyl-reducing enzyme AKR1C was assayed using acenaphthenol as a substrate [42]. Each 0.2 mL assay contained 0.1 M Tris HCl (pH 8.9), 1 mM acenaphthenol, 1 mM NADP^+^ and 10 μL of cytosol. The formation of NADPH in the reaction mixture, which served for the assessment of dehydrogenase activity, was determined spectrophotometrically at 340 nm for 5 min at 37 °C.

The specific activity of CBR1 was assessed in the cytosolic fraction using menadione as a substrate [42]. Each 0.2 mL assay contained 0.1 M K-phosphate buffer (pH 7.4), 500 μM menadione, 250 μM NADPH and 10 μL of cytosol. The consumption of NADPH in the reaction mixture, which served for the assessment of the reductase activity, was determined spectrophotometrically at 340 nm for 5 min at 37 °C. 

The activity of NQO1 was assayed by measuring cytochrome c reduction in the presence of NADH and menadione as an intermediate electron acceptor. The reaction mixture contained 50 mM Tris HCl (pH 7.5) with bovine serum albumin (0.2%), 77 μM cytochrome c, 200 μM NADH, 10 μM menadione, 10 μM dicoumarol (or 0.5% DMSO in the case of uninhibited reaction) and 10 μL of cytosol. The increase in the absorbance of reduced cytochrome c was determined spectrophotometrically at 550 nm for 5 min at 37 °C. The activity corresponding to the NQO1 activity in the cytosol was that which was inhibited by dicoumarol, a known inhibitor of NQO1 [43]. 

The UGT1A activity was assayed in microsomal fraction using *p*-nitrophenol as a specific substrate toward UGT1A [44]. The microsomes (2 mg of protein/mL) were preincubated with a detergent for 20 min at 4 °C, at which time 90 μL of 2.5 mM *p*-nitrophenol, 1 mM UDP-glucuronic acid, 4 mM MgCl_2_, and 100 mM Tris HCl (pH 8.5) were added to 10 μL of the microsomes with detergent. The reaction was stopped after 20 min (37 °C) by 50 μL of 3% trichloroacetic acid. After proper mixing of the reaction mixture, 50 μL of the mixture was added to 50 μL of 1 M NaOH. Control samples were performed in the absence of UDP-glucuronic acid. The absorbance of decreasing substrate *p*-nitrophenol was measured at 405 nm.

The SULT activity assay depends on the formation of 4-nitrophenol and 3′-phosphoadenosine-5′-phosphosulfate from 4-nitrophenylsulfate and 3′-phosphoadenosine 5′-phosphate (PAP) [45]. The reaction mixture contained 5 mM MgCl_2_, 2 μM PAP, 5 mM 4-nitrophenylsulfate, and 100 μM 2-naphthol. After a 10-min incubation of 10 μL cytosol with 90 μL of reaction mixture, the reaction was stopped by 100 μL of 0.25 M Tris-HCl buffer (pH 8.7). Absorbance of 4-nitrophenol was measured at 405 nm. 

Cytosolic GST activity was determined by standard colorimetric assay using reduced glutathione and 1-chloro-2,4-dinitrobenzene (CDNB) as substrates, which was adapted for measurement in 96-well plates [46]. Briefly, each 0.2 mL assay contained 100 mM sodium phosphate buffer (pH 6.5), 1 mM GSH, 1 mM CDNB and 6 μL of the cytosolic fraction. The absorbance of rising product *S*-(2,4-dinitrophenyl)glutathione was detected at 340 nm for 6 min at 37 °C.

### 4.6. Statistical Analysis

All calculations were done using Microsoft Excel and GraphPad Prism 7.03 (GraphPad Software, La Jolla, CA, USA). All values were expressed as mean ± SD. One-way ANOVA was used for the statistical evaluation of differences between the control and treated groups, and differences were considered as significant when *p* < 0.01 (*/+), where * means comparison to the control and + comparison to the BNF treatment. 

## 5. Conclusions

In this study, the activities as well as mRNA expression of drug-metabolizing enzymes were assayed simultaneously to obtain a more complex understanding of sulforaphane’s effect on primary rat hepatocytes influenced and non-influenced by known AhR agonist β-naphthoflavone. SFN alone did not significantly alter the activity and expression of the studied DMEs, except for *Gsta1* mRNA levels, which were significantly enhanced. The known AhR agonist, BNF, markedly induced the activity and gene expression of CYP1A1/2 (both), *CYP3A4* (mRNA), NQO1 (both), CBR1 (both), while the activity of AKR1C and GST and the gene expression of *Sult1a1* and *Ugt1a* were significantly reduced. The co-treatment of hepatocytes with SFN and BNF led to a substantial increase in SULT, AKR1C, CBR1 and NQO1 activity and a marked decrease in *Cyp1a1*, *Cyp2b* and *Cyp3a4* expression. The administration of SFN after BNF induction showed less protective effects against BNF influence on DMEs than the co-treatment of SFN with BNF. Only CBR1, AKR1C and SULT activities were increased compared to BNF-treated hepatocytes. The down-regulation of CYPs, which have been implicated in the bioactivation of various carcinogens [7], and the up-regulation of antioxidant enzymes (e.g., NQO1 and GST) could be important in the prevention of chemical-induced carcinogenesis. Sulforaphane can modulate the DMEs, shifting the balance of the carcinogen metabolism toward deactivation, and this may be an important mechanism of its chemopreventive activity.

## Figures and Tables

**Table 1:** Target and reference genes selected for qPCR. National Center for Biotechnology Information (NCBI) reference sequences, primers, and amplicon sizes.

Gene	NCBI Accession Number	Forward Primer	Reverse Primer	Amplicon Size (bp)
CYP1A1	NM_012540.2	GGGTGGCCTTGAACTCCTTA	TGGTGTAGCGGTTCATGACT	83
CYP1A2	NM_012541.3	CCAACCCAGCCCTCAAGAG	GGATGAGACCACCGTTGTCT	168
CYP3A	NM_013105	GCCCTTTGGAAATGGACCCA	TGCAGAACTTTAGTGAGAGCGA	84
CYP2B	NM_012940.2	CACCAAAGACACCATGTTCCG	TGGTCAAAGTACTGTGGGTCA	99
NQO1	NM_017000.3	TTCCAGCCGACAACCAGATC	AGCCTCCTCCTTTTCCTATCCT	141
CBR1	NM_019170.2	ACCCAAGATGTCTGCAAGGAG	CTGAGACTCACGCTGCTTGAT	83
AKR1C14	NM_138547.3	GGGTTGAAGAGTGTTGCAGG	AAGACCTAGGTTTGGCTCCC	89
GSTA1	NM_031509	CGAAAGCTTTGCAACAATCGC	GCATTAGAAAACGTGTTGGCCT	77
SULT1A1	NM_031834.1	CCTGTCCTTGCTCCCTCAGA	GGAGACAACCACATCCTTTGC	85
UGT1A	NM_201425.2	CCTGGAAATGACTGCCGATG	GCGCATGATGTTCTCCTTGT	82
GAPDH	NM_017008.4	GCAACTCCCATTCTTCCACC	CCACCACCCTGTTGCTGTAG	114
